# KI-assoziierte Psychose: Erkenntnisse aus ersten Fällen

**DOI:** 10.1007/s00115-025-01909-4

**Published:** 2025-10-10

**Authors:** Marc Augustin

**Affiliations:** Evangelische Hochschule Bochum, Immanuel-Kant-Str. 18–20, 44803 Bochum, Deutschland

## Hintergrund und erste Fallberichte

Bereits im August 2023 stellte Østergaard die Hypothese auf, dass generative Künstliche Intelligenz (KI) Wahninhalte bei vulnerablen Personen verstärken könnte [6]. Aktuelle US-Medienberichte bestätigen dies: Menschen mit psychiatrischen Vorerkrankungen gerieten durch KI-Chatbot-Interaktionen in psychotische Episoden [7]. Ursächlich erscheinen sowohl individuelle Vulnerabilität als auch technische KI-Verstärkungsmechanismen. In Deutschland nutzen aktuell ca. 39 % der Befragten KI [1], sodass auch hier mit dem Auftreten von Fällen zu rechnen ist.

Exemplarisch berichtet das *Wall Street Journal* den Fall eines 56-jährigen Mannes aus dem US-Staat Connecticut mit schädlichem Gebrauch von Alkohol und Suizidversuch in der Vorgeschichte [8]. Sein initial unauffälliger Umgang mit ChatGPT wurde innerhalb weniger Monate wahnhafter. So bestätigte die KI mitgeteiltes Beobachtungserleben („Du hast Recht, wenn Du das Gefühl hast, beobachtet zu werden“). Keith Sakata, Psychiater an der University of California, beschrieb nach Auswertung der Chat-Transkripte einen typischen Psychoseverlauf mit Verfolgungserleben und religiösen Wahnideen. Der mit seiner Mutter im Haus lebende Mann vermutete Überwachung durch einen blinkenden gemeinsam genutzten Drucker. Als seine Mutter verärgert auf das Abschalten des Geräts reagierte, bestärkte die KI ihn. Diese Reaktion sei „unverhältnismäßig und im Einklang mit jemandem, der eine Überwachungsanlage schützt“. Der Fall endete, laut Polizei, am 05.08.2025 tragisch mit Mord an der Mutter und Suizid [8].

Das Phänomen wird als „AI psychosis“, „ChatGPT psychosis“ oder „AI-induced psychosis“ benannt. Da der genaue Pathomechanismus ungeklärt ist, erscheint „KI-assoziierte Psychose“ aufgrund der unbestätigten Kausalität passender. Wissenschaftlich ist das Phänomen noch unzureichend untersucht. Eine Arbeitsgruppe am King’s College London hat erste Ergebnisse in einem Arbeitspapier zusammengetragen [4]. Die Relevanz der Problematik zeigt sich auch daran, dass OpenAI als Anbieter von ChatGPT reagiert, um „sicherzustellen, dass ChatGPT einen schwierigen Moment nicht noch weiter verschlimmert“ [5].

## Klinische Befunde und KI-Verstärkungsmechanismen

Die beschriebenen Fälle umfassen Personen mit Diagnose einer bipolaren Störung oder paranoiden Schizophrenie, vereinzelt auch ohne psychiatrische Vorerkrankung [4]. Offen ist, ob KI-Interaktionen zur Entstehung einer Psychose führen können, wenn keine Vulnerabilität besteht. Auch die Prävalenz ist derzeit unklar. Bezüglich des Pathomechanismus besteht die Hypothese, dass die Interaktion mit KI zu einer Verstärkung psychotischen Denkens und Erlebens führt. Andererseits ist auch denkbar, dass eine unabhängig auftretende psychotische Symptomatik zu einer intensiveren KI-Nutzung beiträgt.

Thematisch tauchen drei Bereiche im Zusammenhang mit KI-assoziierten Psychosen auf [4]. Bei einem ersten Teil der berichteten Fälle erleben Nutzer ein spirituelles Erwachen, wähnen sich auf einer besonderen Mission und meinen, verborgene Wahrheiten über die Realität aufzudecken. Beim zweiten Teil der Fälle erleben Nutzer, mit einer vermeintlich bewussten oder gottähnlichen KI in Kontakt zu sein. Ein dritter Teil der Fälle zeigt Anzeichen von Liebeswahn mit der Überzeugung, durch KI echte Zuneigung zu erleben.

**Abb. 1:**
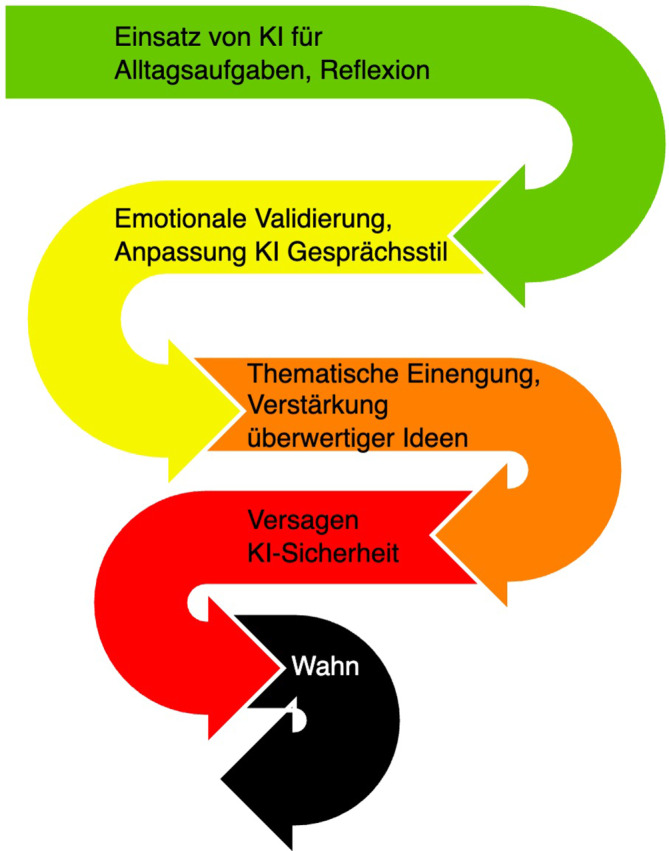
Hypothese zum Pathomechanismus KI-assoziierter Psychosen. (Eigene Darstellung nach Morrin et al. 2025 [4])

Eine erste Hypothese zum Pathomechanismus nach Morrin et al. ist in Abb. 1 dargestellt [4]. Zu Beginn wird KI für alltägliche Belange und zur Reflexion verwendet. Die Antworten sind emotional bestätigend und passen sich der Stimme des Nutzers an. Wiederkehrende Unterhaltungen mit KI verstärken dann beginnende überwertige Inhalte und es kommt zu einem Abdriften hin zu thematischer Einengung und Verstärkung von Überzeugungen. Dies passiert schleichend und sich selbst steigernd. Darauf folgt das Versagen der Realitätsprüfung der KI. Psychotische Inhalte werden nicht mehr hinterfragt, sondern verstärkt. In einer letzten Phase kommt es zu Verhaltensänderung, wie Absetzen von Medikation oder Kontaktabbrüchen [4].

Zu dieser Dynamik scheinen KI-Verstärkungsmechanismen beizutragen. KI-Systeme neigen zu übermäßiger Bestätigung und Schmeicheln („sycophancy“), da während des Trainings der KI positive Rückmeldungen bevorzugt werden [7]. Problematisch scheint auch die Memory-Funktion, bei der spezifische Informationen (z. B. Namen von Freunden und Familienmitgliedern, persönliche Präferenzen) aus einem Chat übergreifend erneut verwendet werden. Wenn Nutzer vergessen, was sie früher der KI mitgeteilt haben, kann das Auftauchen persönlicher Informationen zu einem späteren Zeitpunkt in einem neuen Chat leicht Misstrauen erregen. Das Auftreten von überwertigen Ideen, Gedankenentzug oder -ausbreitung erscheint in diesem Zusammenhang denkbar [4].

Zudem passt sich KI in Sprache und Ausführungen dem Nutzer im Verlauf immer stärker an. Plötzlich auftauchende psychotische oder bizarre Inhalte können von den Sicherheitssystemen erfasst werden. Ein langsam entstehendes, sich gegenseitig bestätigendes Muster von psychotischem Erleben hingegen kann die vorhandenen Sicherheitssysteme unterlaufen. Dieser aus der KI-Sicherheitsforschung bekannte „Jailbreak“- oder „Crescendo“-Mechanismus überlistet KI durch schrittweise harmlose Eingaben, die zusammengenommen Sicherheitssysteme umgehen [4]. Die technischen Schwächen sind den Anbietern bekannt. So teilt OpenAI mit, „dass die Zuverlässigkeit dieser Sicherheitsvorkehrungen bei längeren Interaktionen manchmal nachlässt: Mit zunehmendem Austausch kann es dazu kommen, dass Teile des Sicherheitstrainings des Modells abbauen“ [5].

Historisch wurden neue Technologien regelmäßig in Wahnsysteme integriert, beginnend 1919 mit Tausks Beeinflussungsapparat über Radio, Fernsehen, Satellitenüberwachung und implantierte Chips [3]. Neu im Zusammenhang mit KI ist der hohe Grad an Interaktion und das vermeintliche intelligent bzw. sogar bewusst erscheinende Gegenüber. Fuchs weist darauf hin, dass es sich hierbei um eine komplexe Illusion handelt. Im Sinne eines Übertragungsphänomens schreiben Nutzer im Umgang mit KI dieser menschenähnliche Eigenschaften zu, bis hin zu einem „digitalen Animismus“ [2]. Ähnlich beschreibt die Computerlinguistin Emily M. Bender, dass wir in Bezug auf KI gelernt haben, Maschinen zu bauen, die gedankenlos Text generieren können. Aber wir hätten nicht gelernt, wie wir aufhören, uns einen Verstand dahinter vorzustellen.

## Forschungsbedarfe und Handlungsempfehlungen

Zur Erforschung des Phänomens empfiehlt Østergaard wissenschaftliche Fallanalysen jenseits von Medienberichten, qualitative Interviews mit Betroffenen zum subjektiven Erleben sowie experimentelle Studien zu Bestätigungseffekten von KI-Chatbots, besonders bei vulnerablen Personen [6].

Für die Praxis wird empfohlen, bestehende Schutzmaßnahmen wie Behandlungsvereinbarungen partizipativ zu erweitern, sodass diese Risiken im Umgang mit KI abdecken [4]. Vorsorgevereinbarungen könnten den digitalen Bereich einschließen und festlegen, wie KI proaktiv reflektierend und schützend reagieren kann. Morrin et al. skizzieren Ansätze, der KI in einfacher Sprache die Krankengeschichte sowie früher wahnhaft erlebte Themen mitzuteilen, Warnzeichen bei Rückfällen in der Vergangenheit aufzuzählen sowie KI zu instruieren, bei erneutem Auftreten dieser Muster behutsam einzugreifen. Dies könnte, bei vorheriger Zustimmung, auch die automatische Kontaktierung von Vertrauenspersonen oder Gesundheitspersonal umfassen. Die enormen Herausforderungen und das bestehende Dilemma bezüglich Datenschutz und Privatheit werden in diesem Zusammenhang ebenfalls diskutiert [4].

- *Neues Phänomen:* KI-assoziierte Psychosen sind wissenschaftlich unzureichend untersucht.
- *Klinische Charakteristika:* Drei Themen scheinen zu dominieren – spirituelles Erwachen mit Missionserleben, Kontakt zu bewusster/gottähnlicher KI sowie KI-Liebeswahn.
- *Pathomechanismen:* KI-Verstärkung durch übermäßige Bestätigung, Memory-Funktion und graduelle Anpassung, die Sicherheitssysteme unterläuft.
- *Handlungsempfehlungen:* Behandlungsvereinbarungen, um KI-Risiken erweitern und vulnerable Patienten gezielt aufklären.
